# Sex-Specific Association Between Serum SOD Activity and Osteoporosis in Type 2 Diabetes: A Cross-Sectional Study with Functional Validation

**DOI:** 10.3390/jcm15145601

**Published:** 2026-07-17

**Authors:** Zhuoya Hou, Yanchen Wu, Linlin Zhan, Xinru Du, Ke Xu, Ran Cui

**Affiliations:** 1 Shanghai Tenth People’s Hospital, School of Medicine, Tongji University, Shanghai 200072, China; 2254367@tongji.edu.cn (Z.H.); 2602071@tongji.edu.cn (Y.W.); 2154372@tongji.edu.cn (L.Z.); 2253663@tongji.edu.cn (X.D.); 2 Department of Spinal Surgery, Shanghai East Hospital, School of Medicine, Tongji University, Shanghai 200092, China; 21111290003@m.fudan.edu.cn

**Keywords:** superoxide dismutase, type 2 diabetes mellitus, osteoporosis, bone mineral density, osteoclast differentiation

## Abstract

**Background/Objectives**: Oxidative stress is closely tied to diabetic bone disorders. Superoxide dismutase (SOD) is a key antioxidant enzyme, yet its sex-specific link with osteoporosis in type 2 diabetes mellitus (T2DM) remains unclear. This study aimed to investigate this association and explore its potential biological mechanism. **Methods**: A retrospective cross-sectional study was performed on 2023 T2DM patients (1137 males, 886 females) grouped by bone mineral density (BMD). Multivariate logistic regression was used, and in vitro experiments validated SOD3’s effect on osteoclast differentiation. **Results**: After adjustment for potential confounders, higher serum SOD activity (>135 U/mL) was independently associated with approximately 50% lower odds of prevalent osteoporosis at the lumbar spine and femoral neck in women, whereas no significant association was observed in men. Recombinant SOD3 inhibited osteoclast differentiation in a concentration-dependent way. **Conclusions**: Higher serum SOD activity was independently associated with a lower prevalence of osteoporosis in women with T2DM. Serum SOD activity may serve as a potential biomarker associated with osteoporosis in this population. These findings provide new insights into the relationship between oxidative stress and diabetic bone disease and warrant validation in prospective studies.

## 1. Introduction

Type 2 diabetes mellitus (T2DM) frequently coincides with compromised bone quality, with its underlying causes delved into as β-cell dysfunction, accumulation of advanced glycation end products, inflammatory responses, adipokine alterations and oxidative stress, among others [1]. Notably, oxidative stress holds significant importance in this context. It represents a pathological imbalance between reactive oxygen species (ROS) and the cell’s antioxidant defenses. Elevated auto-oxidative processes and non-enzymatic glycosylation are postulated mechanisms that significantly provoke oxidative stress in T2DM [2]. ROS has been implicated in inducing apoptosis of osteoblasts [3] and osteocytes [4], while also modulating the genesis and lifespan of osteoclasts, thereby favoring osteoclastogenesis and inhibiting mineralization and osteogenesis [5]. Conversely, antioxidants play a pivotal role in maintaining bone mass, either directly or by counteracting ROS actions [6]. Experimental evidence from both in vivo and in vitro studies underscores that antioxidants contribute to activating osteoblast differentiation, enhancing mineralization processes and reducing osteoclast activity, either directly or by mitigating the effects of oxidants [7]. Remarkably, a pronounced decline in plasma antioxidant levels has been observed in both elderly and osteoporotic women [8,9], suggesting that antioxidant status could serve as an indicator reflecting bone mineral density (BMD) in patients with T2DM.

Natural antioxidant enzymes, including superoxide dismutase (SOD), glutathione peroxidase (GPx) and catalase, are instrumental in the degradation of ROS. SOD serves as the cornerstone enzyme in the antioxidant defense system, converting superoxide radicals into hydrogen peroxide and molecular oxygen, which is subsequently metabolized into oxygen and water by additional antioxidant enzymes such as catalase and GPx [10]. Three primary isoforms of SOD exist: CuZn superoxide dismutase (CuZnSOD, also known as SOD1), Mn superoxide dismutase (MnSOD, SOD2) and extracellular superoxide dismutase (ECSOD, SOD3) [11]. Extensive research, both in vivo and in vitro, has established a correlation between SOD and bone metabolism. Specifically, both SOD1 [12] and SOD2 [13] have been implicated in bone loss processes. Notably, SOD1 deficiency is associated with diminished bone stiffness and strength, highlighting its importance in maintaining skeletal integrity [14]. Furthermore, sirtuin 3 (SIRT3) and SOD2 collaborate to regulate bone mass, underscoring their intricate interplay in bone homeostasis [15]. Intriguingly, SOD3 has been shown to promote chondrogenesis in bone marrow-derived mesenchymal stem cells, suggesting its potential role in bone formation and repair [16]. Based on previous evidence, we hypothesized that serum SOD activity may be associated with bone metabolism and osteoporosis in patients with T2DM.

In our previous study, serum SOD activity was identified as a sensitive, convenient, and cost-effective biomarker associated with complications of T2DM [17]. Nevertheless, the association between serum SOD activity and bone metabolism remains poorly understood. To address this knowledge gap, we conducted a retrospective cross-sectional study to investigate the association between serum SOD activity and osteoporosis in patients with T2DM and to explore the biological plausibility of this association through complementary in vitro experiments.

## 2. Materials and Methods

### 2.1. Study Design and Participants

We conducted a retrospective cross-sectional study. Patients with T2DM who visited the inpatient clinic of the Department of Endocrinology and Metabolism, Shanghai Tenth People’s Hospital, Tongji University School of Medicine (China), from August 2009 to November 2023, were reviewed in this study. Study participants were selected based on the inclusion and exclusion criteria. This study was approved by the Ethics Committee of Shanghai Tenth People’s Hospital, Tongji University School of Medicine (approval Nos. SHSY-IEC-4.1/21-41/01, SHSY-IEC-4.1/21-356/01, and SHSY-IEC-5.0/23KY22/P02; approval dates: 26 February 2021, 30 December 2021, and 15 January 2024, respectively). The requirement for informed consent was waived due to the retrospective design of the study.

### 2.2. Inclusion and Exclusion Criteria

Inclusion criteria: (1) newly diagnosed T2DM patients or T2DM patients with diabetes-associated complications (e.g., uncontrolled blood glucose, diabetic complications). Diabetes mellitus was diagnosed by either fasting plasma glucose (FPG) (≥7.0 mmol/L), 2 h plasma glucose (2 h PG) concentration (≥11.1 mmol/L) after a 75 g oral glucose tolerance test (OGTT), or a history of diabetes. T2DM was diagnosed based on the serum insulin or C-peptide level and diabetic-associated antibodies (glutamic acid decarboxylase antibody, insulin autoantibody and islet cell antibody); (2) patients with complete data of BMD; (3) female participants were postmenopausal at the time of enrollment.

Exclusion criteria: (1) patients with acute diabetic complications including diabetic ketoacidosis, hyperosmolar hyperglycemia, infection and so on; (2) patients with severe end-stage renal disease (a history of renal dysfunction or clearance of creatinine less than 30 mL/min) or liver disease (a history of liver dysfunction or alanine aminotransferase more than 100 U/L); (3) acute cardiovascular or cerebrovascular disease (acute coronary syndromes, severe arrhythmology and stroke); (4) patients with malignant tumor; (5) patients on medications that could influence superoxide dismutase (such as alpha-lipoic acid and so on); (6) patients on medications that could influence BMD (such as bisphosphonates, calcitonin and selective estrogen receptor modulator and so on).

### 2.3. Demographic Variables

Medical records on age and sex were collected. Blood pressure, height and weight were documented. Pulse pressure (an index of arteriosclerotic stiffness and impaired arterial conduit according to Windkessel physiology) was calculated as the systolic blood pressure (SBP) minus the diastolic blood pressure (DBP). Body mass index (BMI, kg/m^2^) was also calculated. Information on current medications and menopausal status for women was collected from electronic and paper medical records.

### 2.4. Laboratory Measurements

Laboratory test results were collected from electronic medical records. The methods of testing were as follows. Fasting blood was collected after overnight fasting. Alanine aminotransferase (ALT) and serum creatinine (SCr) were analyzed by a routine auto-analyzer (Modular DP analyzer, Roche Diagnostics, Mannheim, Germany). Total cholesterol (TC) and triglycerides (TG) were determined by the enzyme-linked immunosorbent assay (ELISA) method. To assess creatinine clearance (CrCl) from SCr concentrations measured in units of μmol/L, the Cockcroft-Gault equation was utilized, incorporating gender-specific constants of 1.23 for males and 1.04 for females. Serum SOD activity was measured via autoxidation using the pyrogallol method [18] (Superoxide Dismutase Assay Kit, Beijing Wantai DRD Co., Ltd., Beijing, China), following the manufacturer’s instructions. Briefly, a 15-μL sample was incubated in a 225-μL solution containing Tris-HCl buffer (100 mM, pH 8.2) and 1 mM ethylenediaminetetraacetic acid (EDTA). The samples and buffer were heated up to 37 °C for 5 min. The reaction was initiated by the addition of 75-μL 7 mM pyrogallol. The rate of oxidized pyrogallol (purpurogallin–quinone) formation was incubated at 37 °C for 5 min and then measured spectrophotometrically at 405 nm. The limit of quantification (LOQ) of this assay kit was 15 U/mL. The reference range of SOD is 110 U/mL to 215 U/mL. All biochemical measurements, including serum SOD activity, were performed in the hospital clinical laboratory according to standardized laboratory protocols throughout the study period.

### 2.5. Bone Mineral Density

Values for the areal BMD (g/cm^2^) at the lumbar spine (L1–L4), hip and femoral neck were obtained by DXA on a Hologic QDR 4500 W densitometer with software version 9.03 (Hologic Inc., Bedford, MA, USA). All examinations were performed on one device and by one technologist in the same settings following standardized procedures. The BMD devices provided absolute values for the lumbar spine, hip and femoral neck, along with T-scores or Z-scores. The osteoporosis diagnostic criteria [19] from the World Health Organization in postmenopausal women and men aged ≥50 years were based on the T-score. Normal BMD was defined as a T-score ≥ −1.0. Osteopenia was defined as a T-score from >−2.5 to <−1.0. Osteoporosis was defined as a value for T-score ≤ −2.5 SD in any of the three sites.

### 2.6. Mouse BMM Preparation and Osteoclastogenesis Assay

We isolated primary bone marrow–derived macrophages (BMMs) from the femoral and tibial bone marrow of 6-week-old male C57BL/6 mice. Then, BMMs were cultured in α-minimum essential medium (α-MEM) supplemented with 10% fetal bovine serum (FBS), 1% penicillin/streptomycin and 30 ng/mL macrophage colony-stimulating factor (M-CSF) in a humidified incubator at 37 °C with 5% CO_2_ until approximately 90% confluence. We seeded BMMs into 96-well plates at a density of 6–8 × 10^3^ cells/well, with three technical replicates (triplicate wells) for each treatment group, and cultured in osteoclast induction medium containing 30 ng/mL M-CSF, 50 ng/mL receptor activator of nuclear factor-κB ligand (RANKL), and recombinant SOD3 (0, 1, 2, or 4 μg/mL). The osteoclast culture medium was changed every two days and the number of mature osteoclasts was observed in four groups. Thereafter, the cells were washed twice with phosphate-buffered saline (PBS), fixed in 4% paraformaldehyde for 20 min at room temperature, and then stained using a tartrate-resistant acid phosphatase (TRAP) staining kit. Finally, TRAP-positive cells with more than three nuclei were counted as mature osteoclasts under a microscope. All experiments were independently repeated three times using cells isolated from different mice, and quantitative data were obtained by averaging the triplicate wells from each independent experiment.

### 2.7. Statistical Analysis

Normally distributed continuous data are presented as means ± SD. To compare the patients in three groups including normal BMD, osteopenia and osteoporosis of both sexes, a one-way analysis of variance (ANOVA) was performed when appropriate. Spearman correlation was performed to analyze the association between serum SOD activity and BMD of the sites of lumbar, hip and femoral neck. The association between serum SOD activity and BMD status was further evaluated using ordinal logistic regression analysis. BMD status (normal BMD, osteopenia, and osteoporosis) was treated as an ordinal outcome variable because these categories represent progressively worsening bone health. Therefore, ordinal logistic regression was selected to account for the ordered nature of the outcome and to estimate the association between serum SOD activity and the likelihood of belonging to a lower BMD category. Serum SOD activity level was included as the independent variable. Serum SOD activity was categorized into four grades according to the quartile distribution of the study population. The cutoff values corresponded to the 25th, 50th, and 75th percentiles of serum SOD activity. Accordingly, participants were classified into Grade 1 (≤135 U/mL), Grade 2 (>135 and ≤171 U/mL), Grade 3 (>171 and ≤197 U/mL), and Grade 4 (>197 U/mL). Potential confounding factors included age, BMI, SBP, DBP, ALT, SCr and fasting blood glucose (FPG). Odds ratios (ORs) and corresponding 95% confidence intervals (CI) are reported. Statistical differences were assessed by one-way ANOVA followed by Tukey’s post hoc analysis in vitro experiments. Statistical analysis was conducted in SPSS 26.0 (SPSS Inc., Chicago, IL, USA). A two-sided *p* < 0.05 was considered statistically significant.

## 3. Results

**Figure 1:**
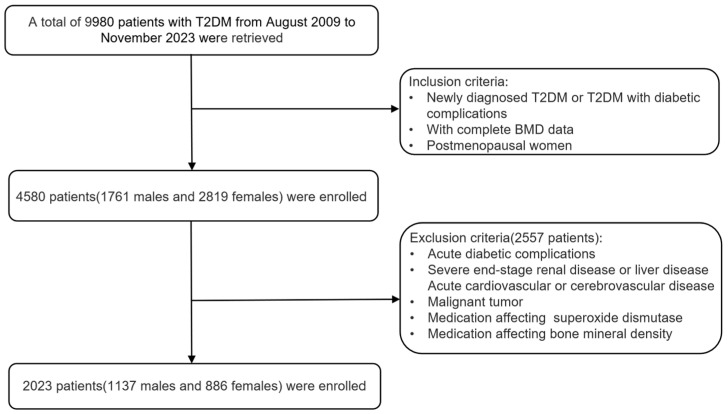
Flow diagram of study participant inclusion and exclusion.

This study ultimately identified 1761 males and 2819 females with T2DM who had complete BMD data. After rigorous screening, 624 males and 1933 females were excluded based on the established criteria. Ultimately, 1137 males and 886 females were enrolled in the study (Figure 1).

### 3.1. Characteristics of the Male and Female Patients

According to the BMD levels, the T2DM patients were grouped into Normal, Osteopenia and Osteoporosis. In males, notable statistical differences emerged across age, BMI, ALT, CrCl and Fasting C-peptide (FCP). In females, significant statistical variations were observed in age, BMI, ALT, CrCl, as well as TG (Table 1).

### 3.2. Comparison of SOD Activities in Groups with Different BMD

Upon analysis, statistical significance in SOD activities was absent among male participants belonging to the normal bone mineral density, osteopenia and osteoporosis categories. Conversely, among females, statistically significant differences emerged across these three groups (Table 1).

**Figure 2:**
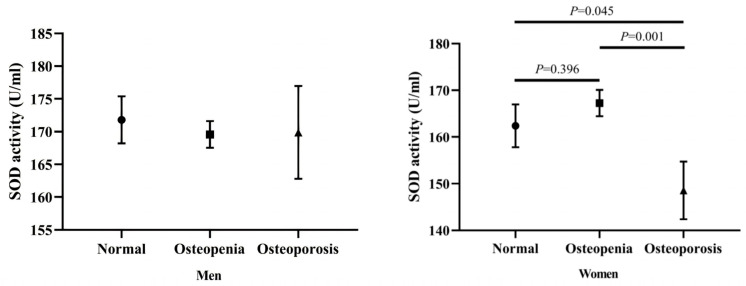
Serum superoxide dismutase activity in different groups in men and women. SOD: superoxide dismutase.

Delving deeper into the female cohort, pairwise comparisons unveiled a consistent trend: individuals within the osteoporosis group exhibited notably reduced SOD activity levels, in comparison to both the normal BMD and osteopenia groups (Figure 2).

### 3.3. Correlation Between SOD Activities and BMD

Upon examination, the correlation between SOD activity levels and BMD at the lumbar spine, hip and femoral neck was not pronounced in the male cohort. In contrast, a significant positive correlation emerged between SOD activity and BMD at the lumbar spine and femoral neck among females, reinforcing the consistency with the aforementioned results (Table 2).

Through a comprehensive application of multivariable ordinal logistic regression analysis, which encompassed variables such as age, BMI, systolic and diastolic blood pressures, ALT, SCr and FPG, we delved deeper into the relationships under investigation. In males, the relationship between SOD activity levels and the prevalence of osteoporosis remained statistically insignificant. However, in females, a significant inverse association was observed between serum SOD activity and the prevalence of osteoporosis at the lumbar spine and femoral neck. Notably, women belonging to the higher SOD activity strata (Grade 2–4) demonstrated approximately 50% lower odds of belonging to a worse BMD category compared with those in the lowest SOD activity group (Grade 1) (Table 3).

### 3.4. Inhibitory Effect of Recombinant SOD3 on Osteoclast Differentiation In Vitro

In the realm of SOD subtypes, SOD1 and SOD2 function as intracellular antioxidant enzymes, whereas SOD3 was distinctively secreted outside the cell. Because SOD3 is the major extracellular isoform of the SOD family and can be administered exogenously in cell culture systems, it was selected as the protein of interest for the in vitro experiments to explore a biologically plausible mechanism related to bone remodeling.

**Figure 3:**
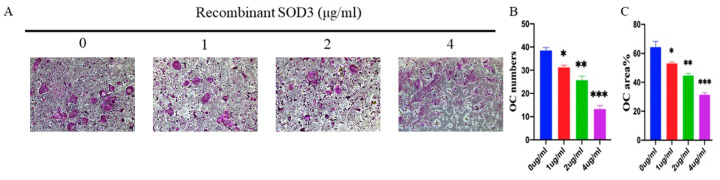
BMMs were treated with different concentrations of recombinant SOD3 followed by the stimulation with M-CSF (30 ng/mL) and RANKL (50 ng/mL). (**A**). Representative images of TRAP staining of BMMs treated with different concentrations of recombinant SOD3 during osteoclastogenesis. (**B**). Quantification of TRAP-positive osteoclast numbers. (**C**). Quantification of TRAP-positive area of osteoclasts. Each treatment group was analyzed in triplicate wells, and all experiments were independently repeated three times. Data are presented as mean ± SD. * *p* < 0.05, ** *p* < 0.01, *** *p* < 0.001 versus the group without recombinant SOD3 treatment.

Primary BMMs were treated in a medium containing RANKL and different concentrations of SOD3 (0, 1, 2 and 4 μg/mL) were added to evaluate the effect of SOD3 on osteoclast differentiation. The number of TRAP-positive multinucleated osteoclasts decreased with the addition of SOD3. Osteoclast area and number of osteoclasts were effectively reduced by treatment with SOD3 in a concentration-dependent manner (Figure 3).

## 4. Discussion

The objective of this study was to elucidate the potential association between SOD activity and BMD in T2DM patients. Through a rigorous cross-sectional analysis, we discerned discernible variations in SOD activity levels across distinct BMD strata. Specifically, among the three cohorts classified by BMD (normal BMD, osteopenia and osteoporosis) the osteoporosis group exhibited the lowest SOD activity levels. Notably, serum SOD activity demonstrated a positive correlation with BMD. To ensure the robustness of our findings, we conducted a multivariate analysis, adjusting for confounding variables such as age, body mass index (BMI), blood pressure, alanine transaminase, creatinine and fasting plasma glucose. In complementary in vitro experiments, recombinant SOD3 inhibited osteoclast differentiation. Because SOD3 is the major extracellular isoform of the SOD family and has been implicated in bone homeostasis, it was selected to explore a biologically plausible mechanism underlying the observed clinical association. The concentrations of recombinant SOD3 used in the in vitro experiments were selected based on previous experimental studies and were not intended to directly replicate physiological serum SOD activity levels observed in patients. Importantly, the in vitro experiments were not designed to replicate circulating total serum SOD activity. Therefore, the in vitro findings should be interpreted as providing biological plausibility rather than direct evidence that serum SOD activity specifically reflects SOD3 activity.

Together with our previous findings that SOD3 promotes osteoblast differentiation and suppresses adipogenic differentiation of bone marrow stromal cells via PI3K/AKT and MAPK signaling pathways through FLT1 modulation [20], the present observation that SOD3 inhibits osteoclastogenesis further suggests that SOD3 may contribute to the regulation of bone remodeling. These findings indicate that SOD3 may influence skeletal homeostasis through both promotion of bone formation and inhibition of bone resorption. This integrated mechanism provides biological plausibility for the observed association between higher serum SOD activity and lower prevalence of osteoporosis in patients with T2DM. Although serum SOD activity was significantly associated with prevalent osteoporosis, its ability to identify individuals at the osteopenic stage remains uncertain. Therefore, serum SOD activity may be more useful as an indicator associated with established osteoporosis rather than early bone loss. Taken together, these findings suggest that serum SOD activity may serve as a potential auxiliary biomarker associated with osteoporosis in patients with T2DM.

Recent studies have predominantly explored the relationship between SOD and bone metabolism from a preclinical perspective. Muthusami et al. reported a notable decline in SOD activity levels within the femur of ovariectomized rats, alongside a robust inverse relationship between hydrogen peroxide production and SOD activity [21]. In *SOD1*(−/−) mice, marked reductions in osteoblast and osteoclast surface areas were observed in lumbar vertebrae [12]. In vitro investigations further revealed that intracellular oxidative stress triggers cell death and impedes proliferation in primary osteoblasts, but not osteoclasts, suggesting that compromised osteoblast viability leads to a decline in osteoblast numbers and subsequently suppresses RANKL/M-CSF-mediated osteoclastogenesis in bone [12]. Morikawa et al. proposed that mechanical unloading, in part, modulates bone mass through intracellular ROS generation and SOD1 expression, implying that SOD1 activation could serve as a preventive measure against mechanical unloading-induced bone loss [22]. Deng’s study consolidated evidence from DNA, RNA and protein levels, supporting SOD2 as a susceptibility gene for osteoporosis [13]. Notably, SOD2 was essential for maintaining monocyte differentiation into functional osteoclasts [23]. In zebrafish models, SOD3 expression was observed in bone tissue and deemed crucial for maintaining normal bone homeostasis and mineralization [24]. However, in the clinical realm, the exact nature of the relationship between SOD and bone metabolism remained to be fully elucidated. To the best of our knowledge, this is the first clinical study to demonstrate a significant inverse association between serum SOD activity and osteoporosis in patients with T2DM. These findings suggest that serum SOD activity may serve as a potential biomarker associated with bone health in this population.

As a potent antioxidant, SOD has garnered widespread application across various disciplines. Its therapeutic potential extends to a broad spectrum of antioxidant-responsive diseases, with inflammation-related conditions, such as enteritis [25], COVID-19 [26], psoriasis [27] and sepsis [28], being among the most prominent. Furthermore, SOD has demonstrated efficacy in managing chronic illnesses, including cardiovascular diseases [29] and Alzheimer’s disease [30]. Our prior research underscores the intricate link between SOD and a range of diabetic complications in T2DM patients, encompassing diabetic macrovascular disease, diabetic peripheral neuropathy and diabetic nephropathy [17]. This study extends the existing evidence by supporting an association between serum SOD activity and bone metabolism in patients with T2DM, bridging the gap between fundamental research and clinical practice at the forefront of the field.

Importantly, a multitude of nutrients have been identified as modulators of SOD activity, hinting at promising strategies for managing T2DM-related osteoporosis. For example, research has demonstrated that drinking hydrogen-rich water effectively reduces alveolar bone resorption in rats subjected to a high-fat diet, which is accompanied by an upregulation of SOD2 and SOD3 genes [31]. Analogously, resveratrol has been shown to significantly bolster osteoblast function through enhancing the expression of antioxidant enzymes, notably SOD3, that protect cells from oxidative damage [32]. Together, the existing body of literature, in conjunction with our findings, supports the potential of serum SOD activity as a translational biomarker associated with diabetic bone health.

One of the most important findings of the present study was the observed sex-specific association between serum SOD activity and osteoporosis. This discrepancy may be attributed to multiple biological mechanisms. First, estrogen plays a pivotal role in regulating oxidative stress and bone metabolism. Postmenopausal women experience a sharp decline in estrogen levels, which promotes reactive oxygen species (ROS) accumulation by weakening antioxidant defense systems while enhancing osteoclastogenesis and impairing osteoblast function, thereby accelerating bone loss [33,34,35]. Consequently, antioxidant enzymes such as SOD may become increasingly important for maintaining skeletal redox homeostasis under estrogen-deficient conditions, making the association between serum SOD activity and osteoporosis more apparent in postmenopausal women. This interpretation is consistent with previous evidence in postmenopausal women. A recent systematic review and meta-analysis of oxidative stress–related biomarkers reported that impaired antioxidant defense was associated with postmenopausal osteoporosis and highlighted a potential relationship between SOD activity and bone health, although substantial heterogeneity existed among individual studies [36]. These findings support a potential role of oxidative stress in osteoporosis and are broadly consistent with the sex-specific association observed in the present study. In contrast, bone homeostasis in men is regulated by multiple androgen-dependent mechanisms. Testosterone and its metabolite dihydrotestosterone (DHT) regulate both osteoblast and osteoclast activity through androgen receptor signaling, and age-related hypogonadism is recognized as one of the major causes of male osteoporosis [34]. Therefore, oxidative stress may represent only one component of the complex regulatory network governing bone metabolism in men. In addition, androgens may help maintain redox homeostasis through multiple antioxidant pathways, including glutathione peroxidase activity [35,37], potentially attenuating the association between serum SOD activity and osteoporosis observed in males. Second, the pathogenesis of osteoporosis differs substantially between sexes. Male osteoporosis is a multifactorial disorder and is more strongly influenced by hypogonadism, smoking, excessive alcohol consumption, reduced muscle mass, and mechanical loading [34,38]. Because these variables were not systematically recorded in our study, residual confounding may also have attenuated the observed association in men. Importantly, the absence of a statistically significant association in men should not be interpreted as evidence of the absence of a biological relationship. Moreover, sex hormone levels were not measured in the present study; therefore, the proposed mechanisms involving estrogen and androgen regulation remain speculative. Future prospective studies incorporating sex hormone profiles, oxidative stress biomarkers, and comprehensive lifestyle data are warranted to further elucidate the mechanisms underlying these sex-specific differences.

Several limitations of this study should be acknowledged. First, the cross-sectional design prevents us from establishing a causal relationship between SOD activity and osteoporosis. Moreover, reverse causation and residual confounding cannot be excluded despite adjustment for multiple potential confounding factors. Prospective cohort studies are required to validate the predictive value of serum SOD for incident osteoporosis in T2DM patients. Second, as mentioned earlier, we did not collect data on factors such as smoking, alcohol consumption, physical activity, vitamin D levels, and parathyroid hormone levels, which may have influenced BMD. Bone turnover markers such as PINP, osteocalcin, and β-CTX were not available and therefore could not be evaluated. In addition, information regarding years since menopause was unavailable in this retrospective study and therefore was not included in the adjusted analyses. Third, the use of antidiabetic medications may have affected oxidative stress levels, and we did not perform stratified analyses based on medication type. Finally, only total serum SOD activity was measured, whereas individual SOD isoforms (SOD1, SOD2, and SOD3) were not quantified. Consequently, the respective contributions of individual SOD isoforms to the observed clinical association could not be determined. Future studies incorporating isoform-specific measurements are warranted. Therefore, the in vitro findings should be interpreted as providing biological plausibility rather than direct mechanistic validation of the clinical observations.

## 5. Conclusions

In conclusion, this large cross-sectional study demonstrated a significant inverse association between serum SOD activity and the prevalence of osteoporosis in female T2DM patients, but not in male patients. Elevated serum SOD activity (>135 U/mL) was associated with approximately 50% lower odds of prevalent osteoporosis at the lumbar spine and femoral neck in women. In vitro experiments further showed that SOD3 inhibited osteoclast differentiation in a concentration-dependent manner, providing mechanistic support for the biological plausibility of the observed clinical association.

Our findings indicate that serum SOD activity may serve as a potential auxiliary biomarker associated with osteoporosis in women with T2DM. Future prospective longitudinal studies are needed to validate these findings, determine the temporal relationship between serum SOD activity and osteoporosis, and further elucidate the underlying molecular mechanisms. Additionally, further investigation into the therapeutic potential of targeting SOD3 for the prevention and treatment of T2DM-related osteoporosis is warranted.

## Figures and Tables

**Table 1:** Clinical features of patients in different BMD levels.

	Men				Women			
	Normal	Osteopenia	Osteoporosis	*p* Value	Normal	Osteopenia	Osteoporosis	*p* Value
Number	392	629	116	—	208	434	244	—
Age (years)	61.3 ± 11.6	62.9 ± 10.2	64.9 ± 9.4	0.003	58.6 ± 10.1	64.2 ± 9.6	70.6 ± 9.4	<0.01
SBP (mmHg)	135.4 ± 19.1	133.4 ± 19.2	132.4 ± 18.5	0.175	132.5 ± 19.1	134.1 ± 19.9	132.0 ± 19.6	0.362
DBP (mmHg)	78.6 ± 12.1	77.2 ± 11.1	77.4 ± 11.2	0.145	78.1 ± 11.1	76.7 ± 10.8	76.3 ± 10.8	0.185
Pulse pressure (mmHg)	56.8 ± 15.1	56.2 ± 15.5	55.0 ± 14.5	0.535	54.4 ± 15.0	57.4 ± 17.3	55.7 ± 17.0	0.085
BMI (kg/m^2^)	25.8 ± 3.5	24.3 ± 3.2	22.7 ± 3.4	<0.001	26.5 ± 3.9	25.1 ± 3.7	23.7 ± 3.6	<0.001
ALT (U/L)	22.9 ± 15.5	19.8 ± 12.4	19.6 ± 12.1	0.001	21.1 ± 13.4	19.2 ± 11.6	17.8 ± 12.0	0.016
SCr (μmol/L)	81.3 ± 25.1	82.3 ± 27.6	77.1 ± 29.6	0.168	61.7 ± 22.2	63.1 ± 19.6	63.9 ± 20.2	0.509
CrCl (mL/min/1.73 m^2^)	100.0 ± 42.7	89.0 ± 31.8	87.1 ± 33.4	<0.001	103.3 ± 38.5	86.9 ± 34.3	72.4 ± 28.6	<0.001
TC (mmol/L)	4.5 ± 1.5	4.3 ± 1.1	4.6 ± 3.4	0.109	5.0 ± 1.4	4.8 ± 1.2	4.9 ± 1.2	0.113
TG (mmol/L)	2.1 ± 2.0	1.6 ± 1.4	1.5 ± 1.1	<0.001	2.0 ± 1.4	1.8 ± 1.4	1.6 ± 1.2	0.043
FPG (mmol/L)	8.5 ± 3.2	8.3 ± 3.2	8.2 ± 3.9	0.607	8.4 ± 3.0	8.4 ± 3.2	8.5 ± 3.5	0.875
FCP (ng/mL)	2.1 ± 1.2	1.9 ± 1.2	1.7 ± 1.1	0.002	2.2 ± 1.2	2.1 ± 1.3	2.4 ± 4.3	0.488
SOD (U/mL)	171.8 ± 49.3	170.6 ± 40.7	169.9 ± 48.1	0.944	162.4 ± 40.0	167.3 ± 37.7	148.6 ± 52.7	0.006

Variables are expressed as means ± standard deviation (SD). — indicates not applicable; *p* values were not calculated for the number of participants in each group. SBP: systolic blood pressure; DBP: diastolic blood pressure; BMI: body mass index; ALT: alanine aminotransferase; SCr: serum creatinine; CrCl: creatinine clearance; TC: total cholesterol; TG: triglyceride; FPG: fasting plasma glucose; FCP: fasting C-peptide; SOD: superoxide dismutase.

**Table 2:** Spearman correlation between serum SOD activity and BMD.

	Men		Women	
	r	*p* Value	r	*p* Value
Lumbar T-score	0.023	0.603	0.127	0.021
Hip T-score	0.063	0.149	0.063	0.257
Femoral neck T-score	0.088	0.051	0.165	0.004

**Table 3:** Ordinal logistic regression between SOD and BMD in men and women.

	Men			Women		
	OR	95% CI	*p* Value	OR	95% CI	*p* Value
Lumbar T-score						
Grade 1	1	—	—	1	—	—
Grade 2	1.184	(0.684, 2.049)	0.546	0.454	(0.245, 0.842)	0.012
Grade 3	1.113	(0.638, 1.939)	0.706	0.519	(0.277, 0.973)	0.041
Grade 4	1.231	(0.697, 2.174)	0.473	0.445	(0.231, 0.859)	0.016
Hip T-score						
Grade 1	1	—	—	1	—	—
Grade 2	1.132	(0.636, 2.017)	0.673	1.304	(0.688, 2.47)	0.415
Grade 3	1.643	(0.921, 2.932)	0.093	1.029	(0.532, 1.990)	0.932
Grade 4	1.291	(0.709, 2.352)	0.404	0.920	(0.462, 1.833)	0.812
Femoral neck T-score						
Grade 1	1	—	—	1	—	—
Grade 2	1.060	(0.582, 1.931)	0.848	0.961	(0.468, 1.974)	0.913
Grade 3	1.213	(0.669, 2.197)	0.524	0.652	(0.317, 1.338)	0.244
Grade 4	1.382	(0.752, 2.541)	0.298	0.470	(0.222, 0.995)	0.049

Grade 1, SOD activity ≤135 U/mL; Grade 2, SOD activity > 135 and ≤171 U/mL; Grade 3, SOD activity > 171 and ≤197 U/mL; and Grade 4, SOD activity > 197 U/mL. Grade 1 was used as the reference category; therefore, the corresponding 95% CIs and *p* values were not applicable. — indicates not applicable. The SOD grades were defined according to the quartile distribution of serum SOD activity in the study population. 95% CI: 95% Wald confidence interval for OR. Potential confounding factors included age, BMI, SBP, DBP, ALT, SCr, and fasting blood glucose (FPG).

## Data Availability

The data presented in this study are available on request from the corresponding author due to privacy and ethical restrictions.
